# Massive gastrointestinal haemorrhage due to gastritis cystica profunda

**DOI:** 10.1186/1757-1626-1-85

**Published:** 2008-08-12

**Authors:** Vinay Itte, Ismail H Mallick, Peter J Moore

**Affiliations:** 1 Department of Gastrointestinal Surgery, Scunthorpe General Hospital, Cliff Gardens, Scunthorpe, UK

## Abstract

**Introduction:**

Gastritis Cystica Profunda is a well recognized entity which may occur several years after previous gastric surgery. This is a premalignant condition and may lead on to carcinoma of the stomach.

**Case presentation:**

We report a case of a 50-year-old man with epigastric pain and haematemesis. 28 years ago he had undergone partial gastrectomy and gastroenterostomy for benign gastric ulcer. An Upper gastrointestinal endoscopy showed a possible bleeding vessel on the anterior wall lesser curve of the stomach. The lesion was injected with adrenaline 1 in 100,000. In spite of the intervention he continued to have haemetemesis with significant haemodynamic impairment. At exploratory laparotomy, an oedematous ridge on the posterior wall with a bleeding point on the posterior gastric wall. Histology showed features consistent with gastritis cystica profunda. He made an excellent post-operative recovery.

**Conclusion:**

We suggest that patients who are diagnosed with gastritis cystica profunda should be regularly followed up as this is a premalignant condition.

## Introduction

Gastritis Cystica Profunda is a well recognized entity occurring several years after previous gastric surgery [1]. It is suggested that ischaemia and chronic inflammation along with the effects of surgery and the presence of suture material may have a role in the pathogenesis of GCP [2]. A correlation seems to exist between gastritis cystica profunda and gastric ulcer [3].

## Case presentation

A 50-year-old man presented as an emergency with epigastric pain and haematemesis. He was continuously vomiting out copious amount of fresh blood. He had a history of heavy alcohol intake and used to consume 10 units of alcohol per day and smoked 20 cigarettes/day. The patient had undergone partial gastrectomy and gastroenterostomy for benign gastric ulcer 28 years ago.

General examination revealed anaemia with no jaundice, clubbing or lymphadenopathy. The pulse rate was 108 beats per minute and blood pressure was 80/50 mm Hg. His laboratory tests revealed Hb 8.9 g/dL and MCV 80.2. Chest and abdominal x-rays were normal. He was transfused four units of packed red blood cells. An urgent upper gastrointestinal endoscopy revealed large amount of fresh blood and clots in the fundus of stomach. A possible bleeding vessel was identified on the anterior wall lesser curve high in the fundus. The lesion was injected with adrenaline 1 in 100,000. In spite of the intervention he continued to have haemetemesis with significant haemodynamic impairment. The patient underwent an exploratory laparotomy which revealed an oedematous ridge on the posterior wall with a bleeding point on the posterior gastric wall.

This was overrun with a Vicryl 2/0 stitch which controlled the bleeding. A biopsy of the oedematous ridge was obtained.

**Figure 1:**
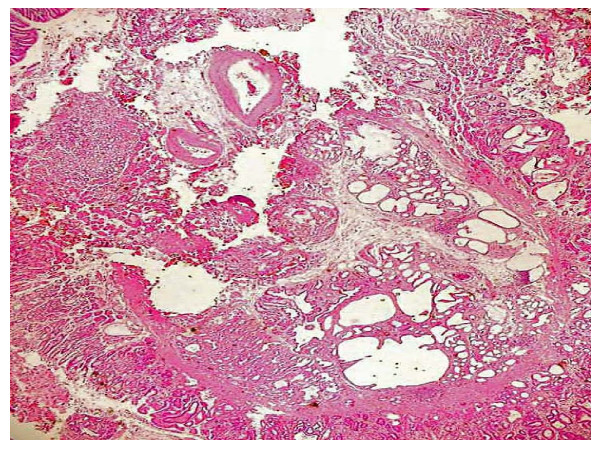
**Histology showing features of gastritis cystica profunda: Widespread disruption of the muscularis mucosa**. The sub mucosa contained numerous glandular structures, lined mainly by antral-type gastric mucosa, showing marked cystic dilatation.

Histology of the oedematous ridge revealed partly duodenal mucosa and partly a mixture of body and antral-type gastric mucosa, the latter showing areas of intestinal metaplasia. There was widespread disruption of the muscularis mucosa. The sub mucosa contained numerous glandular structures, lined mainly by antral-type gastric mucosa, showing marked cystic dilatation. These findings were consistent with gastritis cystica profunda (Fig 1).

The patient was discharged from hospital in ten days without any post-operative complications. Arrangements were made for him to be followed up as an outpatient. He was also advised to stop smoking and consuming alcohol and was referred to support groups.

## Discussion

Gastritis cystica profunda (GCP) is a condition characterized by benign, cystic down growth of gastric glands into the sub mucosa of the stomach [4]. This is a well described entity following previous gastric surgery [1]. The pathogenesis of GCP is probably due to chronic ischaemia and inflammation occurring at the suture site of previous gastroenterostomy [2]. Disruption of integrity of muscularis mucosa causes the migration of epithelial contents into the sub mucosa with subsequent atrophic gastritis, intestinal metaplasia and cystic dilatation of gastric glands [2]. This is a precursor of cancer of the stomach [1]. Gastritis cystica profunda has been described in a patient with a history of gastric ulcer that was treated with H_2 _blockers [5]. This indicates that GCP can occur after exposure of gastric mucosa to chronic mucosal inflammation, which subsequently leads to hyperplastic and metaplastic changes and increased risk for progression towards carcinoma

Littler and Gleibermann proposed that mucosal prolapse and subsequent inflammation play a role in development of GCP [6]. Histologically two stages are evident:

Stage1: with cystic glands limited to mucosal layer (gastritis cystica superficialis).

Stage 2: with gastric glands spreading into the sub mucosa (gastritis cystica profunda).

Gastritis cystica superficialis shows wide cystic glands superficial to muscularis mucosae lined by both columnar and flattened mucous producing epithelium with basophilic cytoplasm [6]. The characteristic feature is the presence of wide cystic glands of pyloric type, together with epithelial lining in the cysts identical to that of crypts. The muscularis mucosae is thickened and further penetration of cystic glands into sub mucosal layer gives them the name gastritis cystica profunda [6], and not dissimilar to colitis cystica profunda where in dilated cystic glands are noted in the bowel wall and this condition has a strong association with inflammatory bowel disease [7].

In their series of 18 patients with GCP, Franzin *et al*. reported that the interval from gastric surgery to the formation of GCP ranged from 3 to 40 years (mean 16.2) and the risk increase with time [8]. In our case report the patient had gastric surgery for gastric ulcer over 28 years ago and presented with severe upper GI bleeding from GCP. There are only three previous cases reported where a patient underwent previous gastric surgery and years later developed haemorrhage from GCP near the anastomotic site [4].

## Conclusion

We suggest that patients who are diagnosed with gastritis cystica profunda should be regularly followed up as this is a premalignant condition.

## Competing interests

The authors declare that they have no competing interests.

## Authors' contributions

VI helped in acquisition of data and preparation of the first draft, IHM was responsible for conception of the idea, overall preparation and revision of the manuscript, PJM was responsible for management of the patient and revising the manuscript for important intellectual content. All authors read and approved the final manuscript.

## Consent

Written informed consent was obtained from the patient for publication of this case report and accompanying images. A copy of the written consent is available for review by the Editor-in-Chief of this journal.
